# Quantifying signal quality in scanning transmission X-ray microscopy

**DOI:** 10.1107/S1600577522004210

**Published:** 2022-05-16

**Authors:** Benjamin Watts, Simone Finizio, Jörg Raabe

**Affiliations:** a Swiss Light Source, Paul Scherrer Institute, Forschungsstrasse 111, 5232 Villigen, Switzerland

**Keywords:** transmission, STXM, uncertainty

## Abstract

While the general effects of experimental conditions such as photon flux and sample thickness on the quality of data acquired by scanning transmission X-ray microscopy (STXM) are widely known at a basic level, the specific details are rarely discussed. This leaves the community open to forming misconceptions that can lead to poor decisions in the design and execution of STXM measurements. A formal treatment of the uncertainty and distortions of transmission signals (due to dark counts, higher-order photons and poor spatial or spectral resolution) is presented here to provide a rational basis for the pursuit of maximizing data quality in STXM experiments. While we find an optimum sample optical density of 2.2 in ideal conditions, the distortions considered tend to have a stronger effect for thicker samples and so ∼1 optical density at the analytical energy is recommended, or perhaps even thinner if significant distortion effects are expected (*e.g.* lots of higher-order light is present in the instrument). (Note that X-ray absorption calculations based on simple elemental composition do not include near-edge resonances and so cannot accurately represent the spectral resonances typically employed for contrast in STXM.) Further, we present a method for objectively assessing the merits of higher-order suppression in terms of its impact on the quality of transmission measurements that should be useful for the design of synchrotron beamlines.

## Abstract 2

Quantifying sources of error in X-ray transmission measurements provides objective guidance for questions of optimum sample thickness and beamline configuration for higher-order suppression.

## 1. Introduction

Many forms of X-ray microscopy, and especially scanning transmission X-ray microscopy (STXM), rely on measurements of the incident and transmitted photon flux in order to determine the absorption of the sample. This can be performed as a function of position for imaging, of energy for spectroscopy, or both for spectro-microscopy, but the nature of the signal remains the same. The quality of the measurement results is well recognized to depend on the signal statistics and the following sections will explore the effects of other experimental parameters such as sample thickness and complicating factors such as dark counts and spectral contamination. This complements previous discussions of X-ray signal quality, normalization, calibration and distortion issues (Nordfors, 1960 ▸; Watts *et al.*, 2006 ▸; Collins & Ade, 2012 ▸), as well as radiation damage (Wang *et al.*, 2009 ▸; Späth *et al.*, 2014 ▸; Berejnov *et al.*, 2021 ▸).

This article is written from the perspective of soft X-ray STXM, but with an effort to keep discussions general in nature. While the physics remains largely the same across the X-ray spectrum (and across techniques applying transmission X-ray measurements), the scale and importance of each effect can vary significantly.

## 2. Signal uncertainty

The precision of a measurement output, such as a spectrum or image, depends on the precision of the measurements it is based on and the way in which they are combined. STXM measurements are typically based on the counting of photons, which follows the statistics of a Poisson distribution. When the count is sufficiently large (≳100) (Lawrence, 2019 ▸), the uncertainty in the number of counted photons, σ_*I*_, is given by the square root of the count, *I*, 

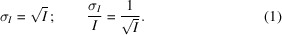

The forward propagation of uncertainty in measured values by their combination in a mathematical formula can be estimated by Taylor series expansion (Ku, 1966 ▸). Some useful forward uncertainty propagation formulas involving the uncorrelated variables, *A* and *B* (whose respective standard deviation is σ_*A*_ and σ_*B*_), include 

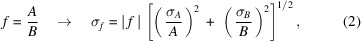




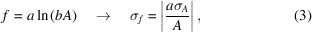




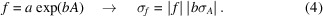

Transmission measurements involve observing the photon flux passing through a sample area, *I*, and comparing it with the incident flux, *I*_0_ (observed via an empty region of the sample), to calculate the optical density, OD, by the Beer–Lambert law,

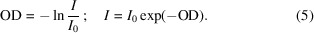

The fractional error in the optical density then follows as

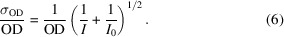

Substituting equation (5) allows the simplification

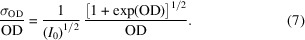

**Figure 1:**
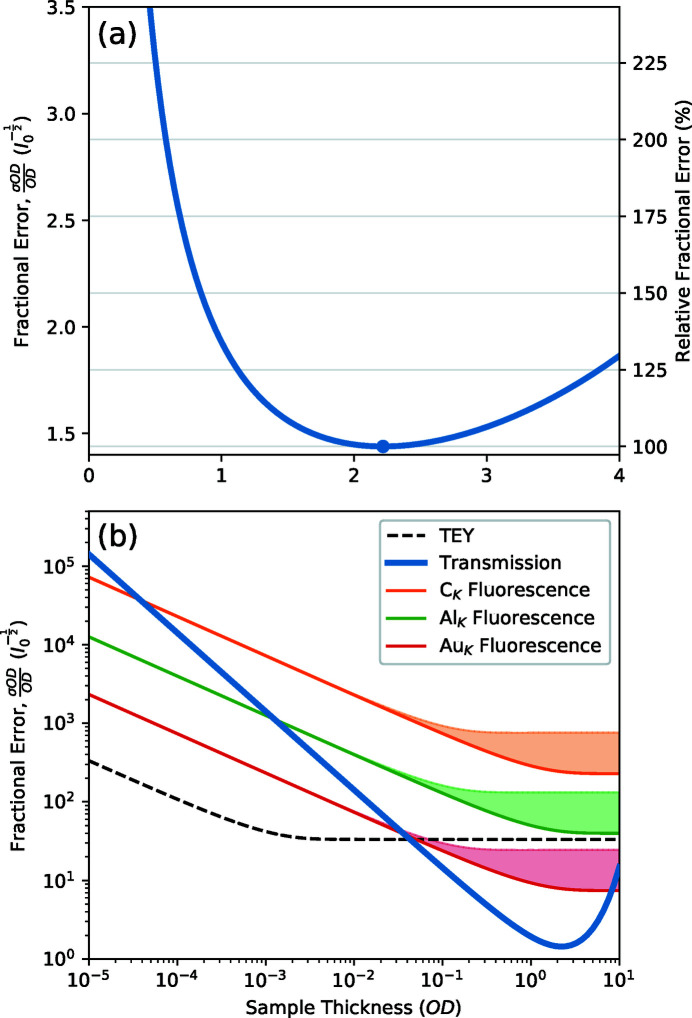
(*a*) The fractional error of a transmission signal plotted as a function of optical density, with the curve minimum indicated by a circle at 2.2OD. The left-hand *y*-scale shows the fractional error in units of 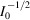 , while the right-hand *y*-scale describes the fractional error as a percentage of the optimum value. (*b*) A comparison of fractional error curves for a transmission signal (blue), as well as fluorescence signals caused by the photo-excitation of the C, Al and Au *K*-edges (orange, green and red curves, respectively, assuming a detector solid angle of 0.24 sr), and an example TEY signal (dashed). Shaded regions indicate the effect of the penetration power of the emitted X-rays decreasing by up to tenfold.

It is reassuring that equation (7) shows an 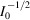 factor to demonstrate that the measured transmission signal quality follows the same Poisson statistics as the component photon count measurements. Fig. 1 ▸(*a*) displays a plot of the second factor of equation (7), separating the effect of sample thickness from that of counting statistics, and corresponds to the fractional error being expressed in units of 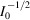 . Interestingly, the curve minimum occurs at 2.2OD (corresponding to 10.9% transmission) and the fractional errors at 1OD and 4OD are both about 130% of the optimum, indicating that high-quality data can be measured with samples much thicker than the typical recommendation of about 1OD. Further, the uncertainty in transmission measurements climbs rapidly as sample thickness decreases below 1OD, meaning that obtaining high-quality results from samples thinner than 1OD requires increasingly higher statistics (*i.e.* counting time).

STXM signals based on the emission of fluorescent photons, or photo-emitted electrons show statistics based on the number of absorption events, *N*_abs_, the probability of relaxation leading to the desired emission type, *P*_E_, the probability that the emitted particle escapes the sample volume, *P*_esc_, and the solid angle of the detector, *A*_D_. Hence, a general ‘emission yield’ (EY) measurement would be composed of 

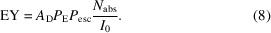

Assuming a homogeneous, flat sample material, an attenuation rate of the sample-emitted signal that is *n* times greater than that of the exciting beam, and a detector positioned on the upstream side of the sample, the detected signal strength is 

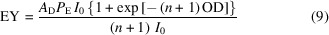

and therefore the fractional error in the emission signal is given by 

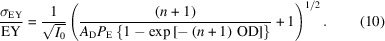

Plots of the fractional error of the fluorescence signals caused by photo-excitation of the C, Al and Au *K*-edges are compared against that of a transmission signal in Fig. 1 ▸(*b*), assuming an *A*_D_ of 0.24 sr [matching the situation at TwinMic (Gianoncelli *et al.*, 2009 ▸)] and *P*_E_ values of 0.001, 0.033 and 0.964, respectively (Schoonjans *et al.*, 2011 ▸). The fractional error of the fluorescence signals displays a weaker dependence on OD than the transmission signal does and so becomes more advantageous for thinner samples. Shaded regions indicate upward movement of the fluorescence fractional error curves as the penetration power of the emitted X-rays decreases by up to tenfold. Electron emission signals are an extreme version of this effect, with the electron signal experiencing an attenuation rate a few orders of magnitude greater than the exciting X-ray beam (Wang *et al.*, 2009 ▸). A fractional error curve for an example total electron yield (TEY) signal is also included in Fig. 1 ▸(*b*), which shows good statistics due to the amplifying effect of the secondary electron cascade, and a wide, flat minimum due to the extremely limited sample depth from which the signal can escape.

Measurements of the incident photon flux, *I*_0_, in a STXM cannot be practically performed in parallel with a measurement because the only part of the X-ray beam path truly representative of the *I*_0_ incident on the sample occurs in the narrow gap between the order selecting aperture (OSA) and the sample surface, which is typically only a few hundred micrometres wide. Hence, measuring the sample and *I*_0_ in series is typically performed. This leaves the experiment susceptible to time-variations in *I*_0_, either by changes in the alignment of the X-ray optics or in the current and orbit of the synchrotron electron beam. Since a high-quality STXM measurement can take many minutes, or even hours, repeating a measurement with the sample removed can result in a significant time delay and hence poor correspondence between the actual and measured *I*_0_ values. The accuracy of the *I*_0_ measurement can be improved by reducing this time delay with an interleaved measurement strategy whereby the spatial regions of the measurement are divided between parts that include the sample material of interest and parts where it is absent. This leads to the question of what proportion of the measurement should be devoted to the *I*_0_ measurement, which can be examined by considering a set of spatial resolution elements (*i.e.* pixels) where *x* corresponds to regions where the sample is absent and are averaged to produce the *I*_0_ measurement, while the remaining (1 − *x*) sample regions are averaged to produce the *I* measurement. Including such statistical proportions in equation (6) gives

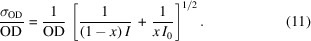

Substituting equation (5) and simplifying then gives

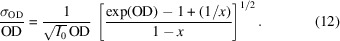

**Figure 2:**
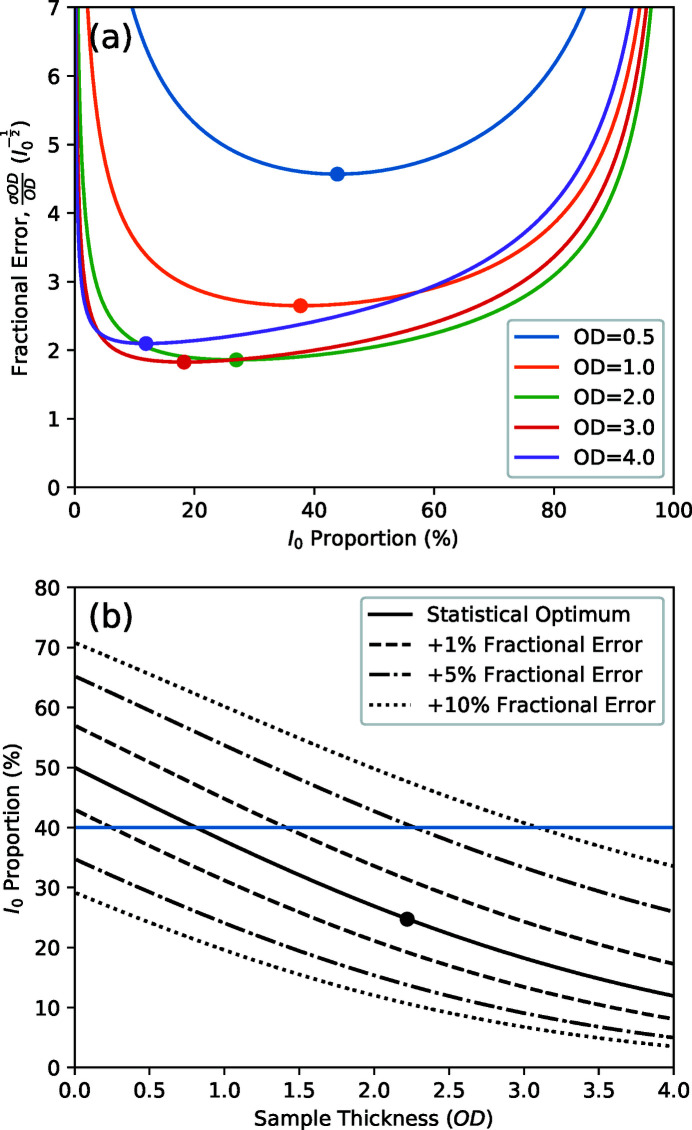
(*a*) The dependence of the fractional error of a measurement on the proportion of the measurement devoted to *I*_0_ for a set of different sample OD values. Circles indicate the curve minima. (*b*) The optimum *I*_0_ proportion for a measurement as a function of sample OD and contours for fractional errors of 1, 5 and 10% above the optimum. The black circle corresponds to a statistical optimum of 2.2OD and 24.7% *I*_0_ proportion, and the horizontal blue line indicates that an *I*_0_ proportion of 40% is a safe choice for a reasonable range of sample OD values.

Fig. 2 ▸(*a*) shows the dependence of a measurements fractional error on the proportion of statistics divided between the sample and incident flux, and the sample OD, as described by equation (12). The optimum *I*_0_ proportion corresponds to the minimum of each curve (indicated by circles) and is observed to move from 50% for very thin samples towards lower *I*_0_ proportions as the sample OD increases. This is in line with the expectations that the statistics are optimized when the *I* and *I*_0_ measurements have an equal number of counts. The curves are observed to have steep walls at the extreme ends of the *I*_0_ proportion scale where either the *I*_0_ or *I* measurement becomes statistically poor, but the middle section shows quite wide and shallow basins where significant changes in the *I*_0_ proportion away from the optimum tend to incur only minor fractional error penalties. This effect can be seen more clearly in Fig. 2 ▸(*b*) where the optimum *I*_0_ proportion is plotted against the sample OD together with contours showing increases of 1%, 5% and 10% fractional error above the corresponding optimum. These contours are surprisingly wide and demonstrate that one has to stray unreasonably far from the optimum *I*_0_ proportion to observe a noticeable degradation of the measurement results. At the statistical optimum sample thickness of 2.2OD, we observe that the optimum *I*_0_ proportion is approximately 25%. However, further exploration of the transmission measurement quality in later sections of this work will provide reasoning for designing samples with OD values lower than the statistical optimum, and an *I*_0_ proportion of 40% [horizontal blue line in Fig. 2 ▸(*b*)] is a reasonable choice for most sample OD values below the statistical optimum, and especially for the range near 1OD.

## 3. Signal distortions

Separate from the statistical issues discussed above, another important consideration in assessing the quality of a measurement is the factors that can distort the measurement results. Such distortions are typically a consequence of the experimental conditions not matching the model by which the data are interpreted and analysed. The corollary of this is that properly including such issues in the data analysis model can mitigate their effects. However, unlike statistical signal-to-noise effects, integrating measurements over longer time periods will not reduce the distortions.

A commonly encountered signal distortion involves an X-ray independent background signal causing an offset to each measurement. These ‘dark’ signals, so called because they are usually observable when the X-rays are blocked with a closed shutter, add a constant value, *d*, to both the *I* and *I*_0_ measurements in a STXM experiment such that the difference between the apparent and actual optical density, ΔOD, is given by

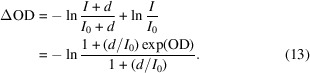

**Figure 3:**
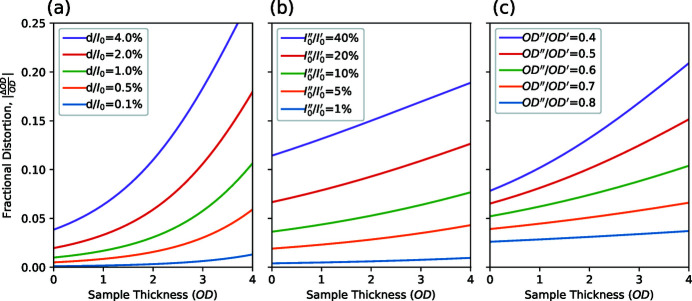
The distortions affecting a transmission spectrum measurement due to higher-order light vary in magnitude depending on (*a*) the presence of dark counts, (*b*) the proportion of higher-order light (with a sample that is twice as transparent to the higher-order light) and (*c*) the relative absorption strength of the first- and higher-order photons by the sample material (with 10% higher-order flux).

Here, we see that the ratio of dark counts to the incident photon flux, 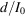 , is a critical parameter in the distortion, with further amplification coming from the OD of the sample. In cases where the *I*_0_ spectrum has significant variation, such as at the carbon *K*-edge where contamination of the optical elements preferentially absorbs the photons (Watts *et al.*, 2006 ▸, 2018 ▸), the relative proportion of the dark signal will vary accordingly and the structure of the *I*_0_ spectrum will be imprinted on the distorted measurement. Fig. 3 ▸(*a*) shows plots of the fractional distortion versus the dark count fraction, 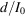 , for a set of common sample OD values. The curves demonstrate how the distortion increases with both increasing dark signal and increasing sample thickness. Note that non-trivial measurements of both images and spectra will necessarily involve variations in the observed sample OD and so the distortion will affect each section of the measurement differently. Since the dark signal is easily measured by simply performing a measurement with the X-ray shutter closed, removing such artefacts is straightforward and effective. However, it is better practice to fix the underlying issues in order to reduce the dark signals to a negligible count rate. While some types of detector have a small rate of background counts inherent to their operation, sources of larger dark signals can often be reduced by simple adjustments of the instrumentation such as shielding the detector from stray light sources (*e.g.* scattered interferometer light, infrared light from position encoders), shielding electrical cables from noise (especially those carrying weak signals), appropriate adjustment of pulse discriminator thresholds, and eliminating electrical ground-loops. In cases where stray light sources inside the experiment chamber form part of the dark signal, it may be observed to vary with the positions of the stages, including the zone plate *Z*-axis that moves according to the photon energy.

A more complicated situation is encountered when the beamline delivers an impure spectrum. An X-ray monochromator will typically pass multiple diffraction orders that allow some photons with integer multiples of the fundamental photon energy to remain in the beam that is delivered to the experiment. This higher-order light is usually more penetrating due to the greater photon energy and so makes the sample appear to have a lower OD. If we consider the observed photon flux to be composed of X-rays from the fundamental (requested) energy, 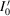 , and another energy, 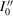 , then the distortion is given by the difference between the actual and intended measurements, 

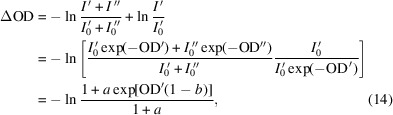

where *a* and *b* are the proportional higher-order light intensity ( 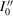 = 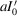 ) and the proportional sample absorption (OD′′ = *b*OD′), respectively, and *d* is the dark signal. While equation (14) does not directly depend on *I*_0_, variations in the 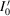 spectrum are unlikely to be matched in 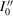 and so will be reflected in the photon energy dependence of *a* and thus help distort the shape of the measured spectrum. Another factor influencing the photon energy dependence of *a* is the detector efficiency, which can be a complicated function of photon energy with many contributing factors. In the soft X-ray range, both phosphor-coupled photomultiplier tubes (Fakra *et al.*, 2004 ▸) and silicon photodiodes (Scholze *et al.*, 1996 ▸; Idzorek & Bartlett, 1997 ▸) typically have detector efficiencies that increase significantly with increasing photon energy and peak at about 800 eV and 1000 eV, respectively. Since the statistics and distortions of a transmission signal only concern the *observed* signal, explicitly deconvolving the detector efficiency and actual photon flux is not necessary for the current discussions. However, the detector efficiency has a direct impact on the radiation dose rate relative to the measurement count rate and so affects the quality of a measurement via radiation damage of the sample (Leontowich *et al.*, 2012 ▸) and carbon deposition (Watts *et al.*, 2018 ▸).

Figs. 3 ▸(*b*) and 3 ▸(*c*) illustrate equation (14) to demonstrate how the signal distortion varies with the proportion of spectral impurity, the relative transparency of the sample, and the sample OD. The similarity of Figs. 3 ▸(*a*) and 3 ▸(*b*) show how higher-order light can produce similar distortions as a simple signal offset (*i.e.* dark signal). However, higher-order light is also much more difficult to characterize sufficiently well to correct its distorting effects due to its greater complexity (*e.g.* varying contributions while scanning the monochromator, varying sample absorption and detector efficiency). In cases where it can be isolated from the main signal well enough to characterize it, removing it to perform the experiment with a more pure beam is nearly always preferable.

## 4. Higher-order suppression

As discussed above, removing higher-order spectral contamination can significantly improve experimental measurements. The basic tools for removing these higher-energy, shorter-wavelength photons from the monochromated beam include transmission filters, reflection filters and specially crafted diffraction structures. Most of these are difficult and expensive to implement and so are often only considered during the design of a beamline. While detailed discussion of the design of such tools is beyond the scope of this work, let us briefly examine the available options in order to encourage greater utilization of higher-order suppression facilities already available. Transmission filters consist simply of a thin membrane (or a gas) made of a material that preferentially absorbs the higher-order photons while remaining relatively transparent to the desired spectral range. These are simple to design and install and so are the most likely type of higher-order suppression to be added to an existing beamline. In the soft X-ray range, transmission filters tend to be effective in removing second-order photons, but not third-order and above since such higher-energy photons tend to be more penetrating. The most effective transmission filters tend to involve a single element with a strong absorption edge just above the desired spectral range, for example second-order light could be removed from the C *K*-edge spectral range by a nitrogen gas filter (Kilcoyne *et al.*, 2003 ▸) (for an attenuation length ratio at 300 eV of 4.1 for first:second order and 1.4 for first:third order) or a thin film of titanium dioxide (for an attenuation length ratio at 300 eV of 2.8 for first:second order and 1.07 for first:third order). At the C *K*-edge, it is common to observe strong reductions in the X-ray beam intensity due to absorption by carbon contamination on the beamline optics (Watts *et al.*, 2018 ▸). In this case, the carbon contamination is acting like a high-pass transmission filter and working to remove it will provide much greater improvements in spectral purity (and additional photon flux) than implementing addition filters.

Reflection filters have a material response aspect similar to a transmission filter, but also a geometrical aspect due to the reliance of X-ray mirrors on total external reflection for efficient beam reflection (Attwood & Sakdinawat, 2017 ▸). The critical angle for total external reflection varies strongly across the soft X-ray spectrum such that lower-energy photons can be efficiently deflected through significantly larger angles than higher-energy photons. Therefore, a strategic choice of both the reflective coating materials and deflection angle of a mirror can very efficiently filter all higher-order photons and can even have an adjustable cut-off through variation of the deflection angle (Frommherz *et al.*, 2010 ▸). Web-based calculators for the efficiency of both transmission and reflection filters are provided by Gullikson (1995 ▸). Since reflection filters change the beam trajectory, they are difficult to incorporate into existing X-ray experiments, especially considering the vacuum and positional stability requirements. Since soft X-ray monochromators are typically based on reflection gratings, the grating itself can be considered a reflection filter and its reflective coating and various line shape parameters are usually optimized to suppress higher-order reflections in particular spectral regions. Plane-grating monochromators often include the ability to adjust the angle of incidence in order to trade between higher-order suppression and overall photon flux via choice of a parameter called the *fixed-focus constant*, *c*_ff_ (Follath & Senf, 1997 ▸). The details of the effects of varying *c*_ff_ will depend on the details of the monochromator design, but most synchrotron beamlines utilizing plane-grating monochromators will have calculations available in their documentation.

While bend-magnets provide a broad, continuous spectrum of synchrotron radiation (as do wigglers to a lesser degree), undulators provide discrete emission peaks (with a fundamental photon energy and higher-order harmonics analogous to the diffraction orders of a grating) that offer some possibilities for higher-order suppression. Firstly, undulators emit their odd-order photons along the beam axis, while the even-order photons are emitted as a hollow cone and therefore the even orders of their emission can be removed by spatial filtering, whereby the beam is passed through an aperture to block the even-order photons (Attwood & Sakdinawat, 2017 ▸). Undulator-based beamlines can also be designed with a quasi-periodic structure in either the monochromator grating (Fujisawa *et al.*, 2001 ▸) or the magnetic lattice of the undulator (Bahrdt *et al.*, 2001 ▸) such that the higher-order harmonics of one are shifted to no longer exactly match the other, and so are not efficiently passed through to the experiment. A similar order-shifting effect can also occur in multilayer gratings (Senf *et al.*, 2016 ▸). As diffractive elements, Fresnel zone plates also have multiple orders and have an effect on the spectral purity of the beam that passes through the OSA. The diameter and positioning of the central stop and OSA should be designed to block the first-order focus of the higher-order spectral components (which will have longer focal lengths in proportion to their higher photon energy), but the *n*th-order focus of the *n*th-order spectral component will follow the same geometry as the first-order focus of the requested photon energy. For a binary zone plate, the even focus-orders are suppressed while the *n* odd orders have a relative efficiency of 1/*n* ^2^ (Attwood & Sakdinawat, 2017 ▸). More complex zone plates can have different focus-order efficiencies that depend heavily on the fabrication parameters (Marschall *et al.*, 2017 ▸).

Quantifying the different types of signal quality provides opportunities to make objective comparisons between them. A commonly considered question in the design of an X-ray beamline and its configuration for a particular measurement is the level to which higher-order light should be suppressed versus the resulting loss of first-order photon flux from the use of transmissive or reflective filters. If we consider the statistical variance and distortions described in equations (7) and (14) to be of equal significance, then we can combine the two into a measure of total signal error, *E*_T_,

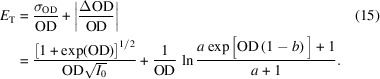

**Figure 4:**
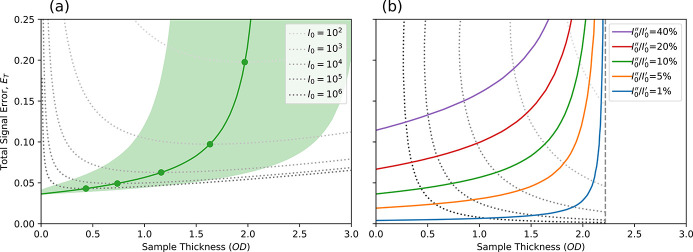
(*a*) Signal quality curves for 10% higher-order light (absorbed at a 0.6 relative rate) and varying levels of counting statistics are shown as dotted grey-scale contours and minima positions labelled with green circles. The solid green curve shows the trajectory of the minima and the shaded region indicates the sample OD range corresponding to an increase of signal error of up to 10% above the minima. (*b*) Curves of optimum signal quality corresponding to measurements with higher-order contributions shown in Fig. 3 ▸(*b*). The corresponding statistics contours are shown as dotted lines, while the vertical dashed line shows the optimum sample OD of 2.2 for ideal measurement conditions.

Plots of such total signal error curves calculated for an experiment in realistic conditions that involve a 10% higher-order light contribution (that is absorbed at a rate of 0.6 times that of the main X-ray energy) are shown as grey-scale dotted lines in Fig. 4 ▸(*a*) for varying levels of incident photons per resolution element. Green dots show the minima of the error curves and the solid green line describes the trajectory of the optimum sample thickness as a function of the scale of statistics. Note that the total signal error curves have quite broad minima and the light green shaded area describes the range of sample thicknesses that lie within 10% of the optimum signal quality. This range is relatively broad and indicates that, when working with an impure probe beam, a sample thickness of about 1OD should work reasonably well for most statistical levels. Fig. 4 ▸(*b*) shows a wider view of the balance between statistical and distortion issues by calculating the signal error mimima curves for different levels of the higher-order light contributions. [Note that the legend of Fig. 4 ▸(*b*) matches that of Fig. 3 ▸(*b*).] The dotted lines in Fig. 4 ▸(*b*) demonstrate the points at which the aforementioned optimum sample thickness curves intersect the total signal error curves at a specific level of counting statistics. These two sets of curves illustrate how consideration of the optimum sample thickness weighs the effects of noise against signal distortions, with each starting close to an OD of about 2 in the low-statistics region and shifting towards lower OD with improving statistics at rates determined by the severity of the distortions caused by the experimental conditions. This trend in Fig. 4 ▸(*b*) demonstrates that thinner samples (*e.g.* ∼0.5OD) are recommended for measurements at instruments with poor spectral purity and higher photon flux, while thicker samples (*i.e.* 1–2OD) are recommended for use with instruments with high spectral purity and/or lower flux. The fact that every line plotted in each part of Fig. 4 ▸ does not meet the origin also demonstrates that very thin samples cannot completely eliminate distortions and that the presence of spectral impurity puts a hard limit on the achievable quality of transmission measurement results.

**Figure 5:**
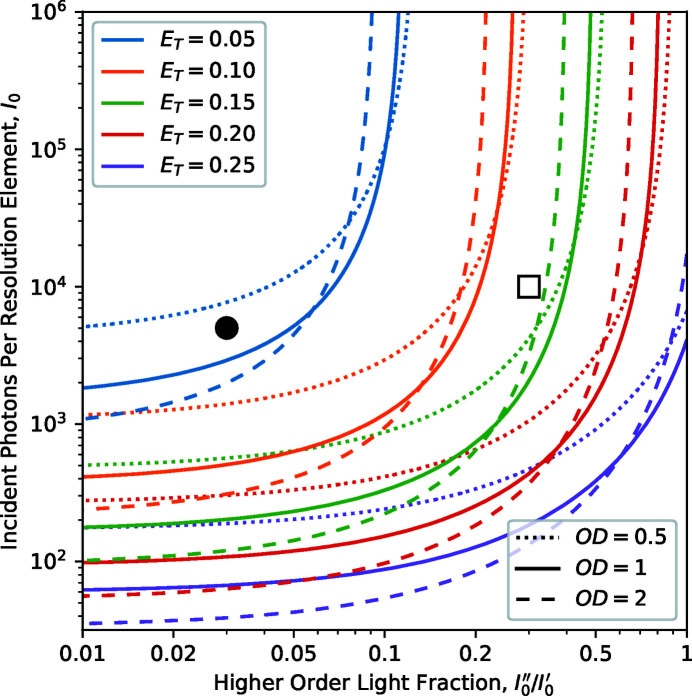
A contour map of total signal error, *E*_T_, as a function of incident photons per resolution element, *I*_0_, the higher-order light contribution [parameter *a* from equation (14)] and the sample OD. The black markers indicate the conditions for C *K*-edge measurements at the PolLux beamline with (filled circle) and without (empty square) the higher-order suppressor.

Fig. 5 ▸ illustrates equation (15) from a more general perspective via a contour map of *E*_T_ values resulting from the measurement conditions in terms of statistics, higher-order light, and sample OD. It is clear that compared with the set of contours for OD = 1 (solid lines), the OD = 2 dashed contours tend to be shifted down and to the left, while the dotted OD = 0.5 contours are shifted up and to the right, demonstrating the statistics advantage of thicker samples and the resilience against distortion by thinner samples. Operating the PolLux beamline at the carbon *K*-edge (∼300 eV) without the higher-order suppression mirror system (Frommherz *et al.*, 2010 ▸) will result in a higher-order light contribution of about 30% and about 10 MHz of first-order photon flux, which translates to an *I*_0_ of about 10^4^ with 1 ms of counting time per scan point. This situation results in an *E*_T_ value of about 0.13 and places the measurement towards the right hand side of Fig. 5 ▸, as marked with an open square symbol. Applying the higher-order suppressor with a mirror angle of 1.5° will reduce the 300 eV flux by about half while reducing the higher-order fraction by about an order of magnitude (Frommherz *et al.*, 2010 ▸). This set of conditions is indicated in Fig. 5 ▸ by a filled circle symbol and the improvement in transmission measurement performance is illustrated by the 0.1 and 0.05 *E*_T_ contours lying between the two symbols. Further, the filled circle is now toward the left side of the plot where the contours have a lower gradient; from this position, an increase in statistics through increasing the counting time would move our position vertically upwards into lower-*E*_T_ regions near the upper left corner of the figure for further improvements in measurement quality. In contrast, the position without higher-order suppression (open square symbol in Fig. 5 ▸) is surrounded by almost vertical contours and so any increase in counting time would not provide a significant improvement in measurement quality under these conditions.

## 5. Spatial resolution

**Figure 6:**
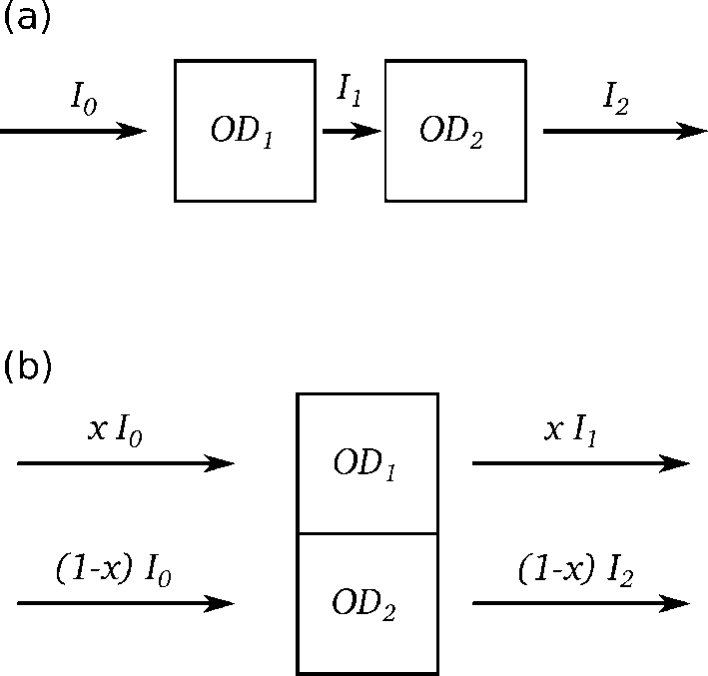
Schematic of an X-ray beam transmitting through two materials, OD_1_ and OD_2_, in (*a*) series and (*b*) parallel with a fraction, *x*, of the beam passing through OD_1_ and the remaining fraction, (1 − *x*), passing through OD_2_.

Microspectroscopy investigations are often concerned with measuring the spectrum of small sample targets. To properly isolate the target area, one must make sure that the spatial resolution, focusing and positioning of the probe beam are all sufficient. This can be judged by examining how well resolved the target object appears in an image scan. In cases where the illuminated area is larger than the spatial separation of sample materials, the beam will interact with materials (or lack thereof) adjacent to the target and the resulting spectrum will be some mixture of the spectra of the intended target and the other materials that intercept the X-ray beam. Fig. 6 ▸ shows schematics of an X-ray beam passing through two materials, OD_1_ and OD_2_, in series or parallel. Let us first examine the series case from Fig. 6 ▸(*a*) where the sample materials are stacked along the direction of the beam axis and so the spatial resolution is not relevant. Applying the Beer–Lambert law to these materials individually gives

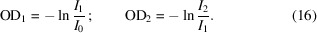

However, in practice it is typically not possible to measure *I*_1_ and we are restricted to interpreting the analysis of measurements of *I*_0_ and *I*_2_. This fortunately turns out to simply give the sum of the two component spectra,

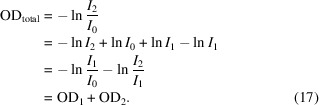

This works out neatly because a basic property of logarithms is that the logarithm of a product is equal to the sum of the logarithms of the factors. On the other hand, the analysis of a beam passing through two materials in parallel, as shown in Fig. 6 ▸(*b*), does not provide the same kind of eloquent result since there is no corresponding property for the logarithm of a sum. If the two materials and their Beer–Lambert law interactions with an X-ray beam are given by 

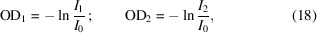

then let us examine the amount of distortion, ΔOD, in a measurement as the difference between the actual analysis (logarithm of combined beams) and the naïve expectation of the weighted sum of the component material spectra, OD_1_ and OD_2_, proportional to the corresponding fraction of the X-ray beam, *x* and (1 − *x*), passing through each material component, 

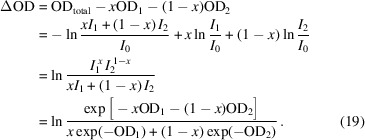

**Figure 7:**
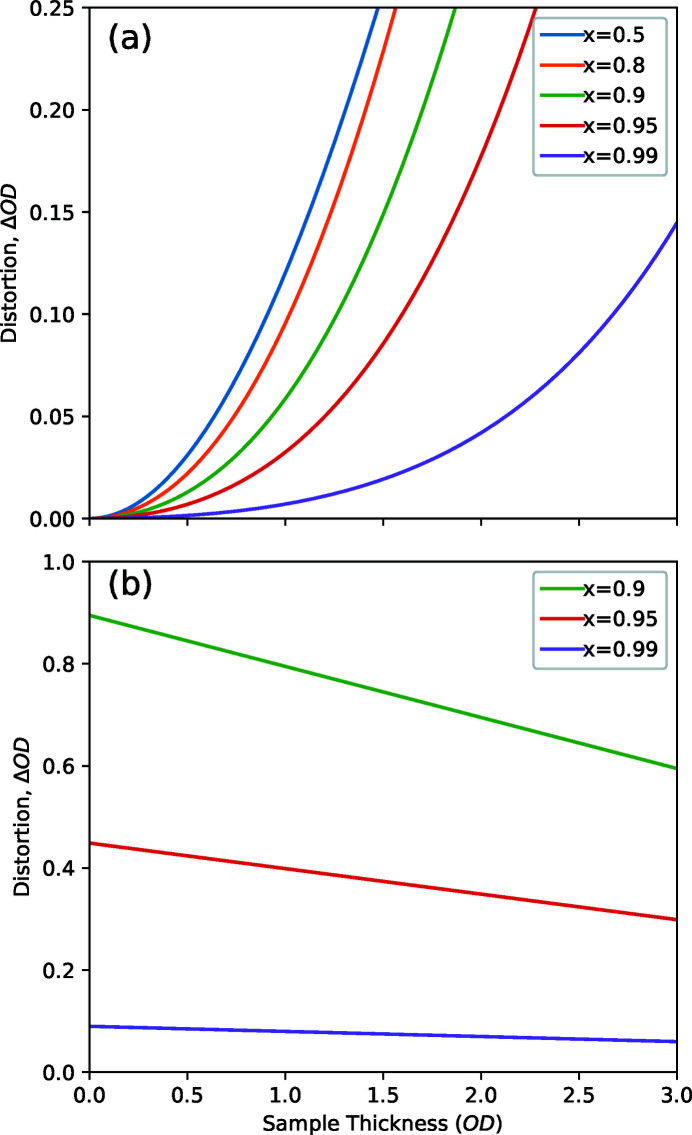
Distortion estimates for situations where only a fraction of the beam (given by *x*) intercepts the intended sample material and the remaining beam goes through (*a*) empty space (*i.e.* 0OD) or (*b*) a strongly absorbing object (10OD).

Equation (19) only gives zero for the trivial cases where *x* = 0, 1 or OD_1_ = OD_2_ and so some kind of distortion should always be expected in transmission measurements where some part of the probe beam falls outside the target area; for example, measuring the spectrum of a nanoparticle with a poor focus. Fig. 7 ▸(*a*) illustrates these distortions as a function of the optical density of the target material, OD_1_, and for different fractions of the probe beam intercepted by the target, *x*, in the situation where the probe beam partially goes through a hole in the sample (*i.e.* OD_2_ = 0). Here we observe a series of parabolas that indicate a distortion term that is roughly proportional to the square of the target optical density. The other extreme case of a thick object (OD_2_ = 10) blocking part of the beam is demonstrated in Fig. 7 ▸(*b*) and this time we observe a set of roughly horizontal lines, indicating that the measured spectrum differs from the target spectrum by an approximately constant offset. These results tell us that the expected linear combination of parallel-mixed transmission measurements is overshot by one term in each direction, with the lower OD component receiving a roughly constant offset and the higher OD component receiving a quadratic scaling factor.

It is important to note that the above discussion of parallel transmission measurements applies to all kinds of sample inhomogeneity that create contrast in the transmission signal; chemical, dichroic, thickness or density fluctuations with a transverse length scale below the beam spot size will all cause an imperfect averaging of the component spectra. The concept of thickness/density fluctuations causing spectral distortions in an otherwise homogeneous material is surprising, but equation (19) applies to any situation where the unresolved sample regions have non-identical OD at any photon energy involved in the measurement of a single resolution element. One should therefore be wary of transmission spectra measured from an agglomeration of particles, or from very rough, highly porous or irregularly shaped samples that are not spatially resolved since the mixture of different optical paths can introduce distortions. Another situation applicable to equation (19) is the measurement of a very thick sample in the neighbourhood of a large open area, such as across the edge of a thick film or microtome slice, where the outer side-lobes of the zone plates illumination can result in a small fraction of the probe beam going around the sample material. This could correspond to the red, *x* = 0.95, line towards the right edge of Fig. 7 ▸(*a*) where significant distortion can be expected with very little extraneous light. Marcus *et al.* (2021 ▸) recently examined similar situations in detail and demonstrated how the finite point spread function of a zone plate can distort conventional STXM measurements in the neighbourhood of an interface such that it differs from a linear combination of the component spectra. However, they do not consistently differentiate between the simple mixing effects of a finite beam–sample interaction volume and the distortions caused by inequivalent optical paths. Further, they overstate the severity of the effect by using the very unrealistic and impractical example of measuring a 2 nm-thick polymer [which equation (7) shows to be a challenge in itself] at an interface with a material 100 times thicker. It is unfortunate that they do not mention probe deconvolution (Jacobsen *et al.*, 1991 ▸; Loroña Ornelas *et al.*, 2018 ▸) which is a post-processing method that mitigates such effects by directly accounting for the finite size of the probe. Note that the probe deconvolution must be performed on the transmission data (*i.e.* before converting to OD) in order to avoid the distortions described by equation (19).

The central stop and OSA components of a STXM work together to block the direct (zero-order) X-ray beam. Poor size-matching or alignment of these components, as well as materials not thick and dense enough to be opaque, would allow a significant, broad-area beam to surround the focused beam and produce situations corresponding to Fig. 7 ▸ with low *x* values, depending greatly on the sample OD over a wide area (similar to the OSA diameter). Fabricating the central stop for a zone plate presents a technical challenge that is often un­appreciated; an integrated central stop adds stress to the zone plate’s support membrane and so the thickness of the stop must be compromised to avoid breakage. On the other hand, a separate central stop requires extra positioning stages and regular effort to keep properly aligned.

Interestingly, cases where the spectral resolution is poor relative to the gradient of the samples absorption spectrum also count as a mixture of inequivalent optical paths and so equation (19) is relevant to understanding the measurement conditions. Consider an X-ray beam composed of two photon energies, illuminating the same area of a homogeneous sample and experiencing different absorption rates due to the component photon energies being on and next to a sharp absorption resonance. Therefore the two beam components would see different OD values in the same sample and, if we call these OD_1_ and OD_2_, and their flux proportions as *x* and (1 − *x*), then the error in assuming that the parallel measurement results in a weighted average is already stated in equation (19). Utilizing equation (19) to understand experiments conducted with an X-ray beam in which two photon energy components differ by a factor of two or three illustrates how it can also be considered as an alternative view of the distortions caused by higher-order light.

## 6. Sample thickness

So far we have restricted discussion of the sample to OD, which is independent of the sample composition, density and thickness, as well as the photon energy of the X-ray beam. However, practical application of the topics discussed above requires an understanding of how these parameters interact. Generally, the OD of a sample increases with increasing thickness, density, higher atomic number elements, and longer X-ray wavelengths (decreasing photon energy). While the sample OD is simply proportional to the material thickness and density, its dependence on other parameters can be complex. Optical density is defined as the degree to which a medium reduces the transmission of light, as expressed in equation (5) with 1OD being equivalent to a reduction to 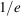 of the original intensity. This matches the *attenuation length* of a material, defined as the path length through a material that will reduce light to 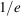 of the original intensity. (Note that the *attenuation length* is also the reciprocal of the *linear attenuation coefficient*.)

**Figure 8:**
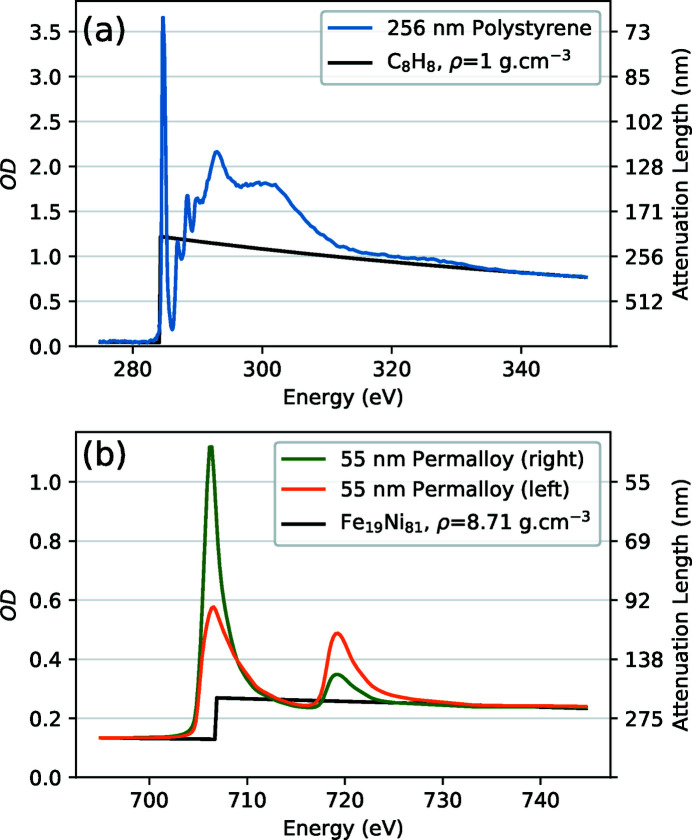
Comparisons between experimental transmission measurements of films of (*a*) polystyrene and (*b*) permalloy (Fe_19_Ni_81_) and corresponding calculations based on the Henke scattering factors (Henke *et al.*, 1993 ▸) and material parameters. The left-hand *y*-axis scale is presented in OD, while the right-hand *y*-axis scale displays the corresponding attenuation length.

An estimation of a material’s attenuation length can be calculated from published atomic scattering factors (Henke *et al.*, 1993 ▸) together with its elemental composition and density. Such calculations can be conveniently performed with online calculators (Gullikson, 1995 ▸; Chantler *et al.*, 2019 ▸). However, it is important to be mindful that these calculated values do not include any of the near-edge fine structure, as illustrated in Fig. 8 ▸. Fig. 8 ▸(*a*) compares the measured C *K*-edge spectrum of a 262 nm-thick polystyrene film with a spectrum calculated from the Henke *et al.* (1993 ▸) scattering factors using the chemical formula C_8_H_8_ and a mass density of 1 g cm^−3^. The calculated spectrum fits the measurement closely in the pre-edge (below 282 eV) and post-edge (above 320 eV) regions, but the near-edge resonances differ significantly from the calculation. It is important to consider the near-edge resonances since they are typically targeted in measurements in order to provide contrast between constituent sample materials, or to indicate physical parameters like magnetization or molecular orientation (Ade & Stoll, 2009 ▸; Watts & Ade, 2012 ▸). The *y*-axis scale on the right hand side of Fig. 8 ▸ reflects the attenuation length corresponding to the OD values shown in the *y*-axis scale to the left. The 256 nm-thick polystyrene film matches 1OD for measurements at 320 eV (*i.e.* polystyrene has an attenuation length of 256 nm at 320 eV), but the 1*s* 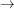 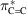 resonance near 285 eV that peaks at 3.66OD in this measurement would require a sample thickness of 70 nm in order for this point in the spectrum to be measured as 1OD. When trying to obtain contrast between polystyrene and other organic materials, the unusually intense 1*s* 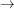 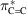 resonance near 285 eV is naturally a common choice to include in the set of photon energies to be measured as it often provides strong contrast; however, it would be wise to limit the sample thickness in such experiments to ∼100 nm (or perhaps less) in order to limit the polystyrene 1*s* 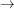 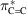 resonance peak to about 1.5OD and hence limit the associated signal distortion issues. In cases where the ability to choose the sample thickness is limited, one could consider instead performing the analysis with weaker resonance peaks.

Fig. 8 ▸(*b*) compares X-ray magnetic circular dichroism (XMCD) measurements of a 50 nm-thick permalloy film (in the presence of a 3 T out-of-plane magnetic field) with a spectrum calculated from the Henke *et al.* (1993 ▸) scattering factors using the chemical formula Fe_19_Ni_81_ and a mass density of 8.71 g cm^−3^. Note how the Fe *L*_2,3_ near-edge resonances differ significantly from the calculated spectrum and also vary in intensity depending on the circular polarization of the X-ray beam. Other kinds of samples can display linear dichroism and display a similar variation in peak intensity depending on the alignment between the transition dipole moment of the resonance and the electric field vector of the linearly polarized X-ray beam. The thickness of the permalloy film shown in Fig. 8 ▸(*b*) was chosen such that the maximum observed Fe *L*_3_ resonance would be approximately 1OD in order to limit signal distortions caused by insufficiently opaque central stops. An estimate of the desired sample thickness, *t*_sample_, in order to measure a specific resonance at a chosen intensity, OD_target_, can be calculated by comparing the relative OD of the target resonance with a reference point in a nearby spectral region without significant resonances, 

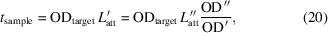

where OD′, OD′′, 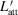 and 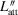 are the absorption intensity and attenuation length corresponding to the target resonance and reference point, respectively. It is recommended to choose the reference point in the post-edge region [*e.g.* at 340 eV and 740 eV in Figs. 8 ▸(*a*) and 8 ▸(*b*), respectively] where scattering factor-based calculations tend to be accurate and the higher absorption rate provides better precision than the pre-edge region. Since the calculation uses a ratio of OD values, the scaling of the spectrum does not affect the result, but a background subtraction (often used to set the pre-edge to zero) will. Note that spectra measured by other detection modes such as total, partial and Auger electron yield (TEY, PEY and AEY, respectively) or fluorescence yield (FY) are not exactly the same as transmission measurements. For example, AEY and FY modes where the observed signal is specific to a particular element and electron shell (*i.e.* only accept specific emission lines) should give zero intensity in the pre-edge region, and so are equivalent to a transmission spectrum after a background subtraction.

Knowing the strength of a resonance for a material one has not yet measured presents the challenge of finding a suitable reference spectrum. Since theoretical calculations of NEXAFS spectra tend to be unreliable for lighter elements, searching the literature for previously published measurements is often a better strategy. Fortunately, the NEXAFS spectrum of a mixture is typically equal to the linear combination of the component spectra (Stöhr, 2013 ▸) and so a reference spectrum does not need to exactly match the planned sample material in order to be useful for a thickness estimate calculation. For organic materials, intermolecular effects on NEXAFS spectra are subtle and rarely observed (Zou *et al.*, 2006 ▸), while intramolecular effects causing departures from the *building block* model can be more significant, but still rarely observed (Stöhr, 2013 ▸; Watts *et al.*, 2011 ▸). Reference spectra can often be quickly found by searching the internet for images related to the material, elemental edge of interest and the spectroscopy type; for example, ‘polystyrene C *K*-edge NEXAFS’ and ‘permalloy Fe *L*-edge XMCD’ will return results similar to Fig. 8 ▸. Note that near-edge X-ray absorption fine structure (NEXAFS) and X-ray absorption near-edge structure (XANES) spectroscopies are two names for the same technique that analyses the resonance peaks that occur close to an absorption edge, but extended X-ray absorption fine structure (EXAFS) is a very different analysis that examines oscillations extending into the far post-edge region (Norman, 1986 ▸).

## 7. Conclusions

We have shown that, in addition to the 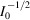 dependence expected from Poisson statistics, the statistical noise of transmission measurements in ideal conditions is minimized for a sample with a thickness of 2.2OD (although this minimum is quite shallow, with sample thicknesses between 1.4 and 3.2OD resulting in an increase of fractional error of less than 10%). This is surprisingly thick, corresponding to only 10.9% transmitted intensity, compared with the conventional wisdom that transmission samples should be about 1OD thick. On the other hand, we clearly observe that signal distortions due to dark signals, higher-order spectral contributions and insufficient resolution are magnified in thicker samples. By summing contributions from the statistical and distorting effects, we obtain an objective measure for the quality of results to be expected from a transmission measurement and map out the dependence of optimum sample thickness on the applied number of photons and their spectral purity. We found that the spectral purity of the probe beam is a major factor in determining the optimum sample thickness, which will be closer to 1OD at many instruments where more than a few percent of higher-order light is expected and even ∼0.5OD in cases of very poor spectral purity and high photon flux. Overall, these results support the conventional wisdom that a sample thickness of ∼1OD at the analytical energy is a safe choice that will provide near-optimal results in most experimental conditions. While 1OD is equivalent to a sample thickness equal to the attenuation length of the material, one must keep in mind that simple calculations of attenuation length based on the elemental composition and density of the sample material will not include the near-edge resonance peaks that are often targeted in experiments. We therefore present a simple method for estimating the attenuation length corresponding to a near-edge resonance peak.

We further present a generalized map of the transmission signal quality as a function of the higher-order light fraction and the applied counting statistics and then use it to demonstrate how the application of the higher-order suppressor mirrors improve the quality of C *K*-edge measurements at the PolLux STXM. This type of map could be useful in the design of X-ray beamlines, and the planning of experiments, as an objective measure of the consequences of higher-order suppression.
